# Experimental Study of Wear Behavior Under Friction for Fused Filament Fabrication Components

**DOI:** 10.3390/ma19081575

**Published:** 2026-04-14

**Authors:** Marius Bădicioiu, Răzvan George Rîpeanu, Cristina Maria Dușescu-Vasile, Mihaela Mădălina Călțaru, Alexandra-Ileana Portoacă

**Affiliations:** 1 Mechanical Engineering Department, Petroleum-Gas University of Ploiesti, 100520 Ploiești, Romania; mbadicioiu@upg-ploiesti.ro (M.B.); rrapeanu@upg-ploiesti.ro (R.G.R.); miki@upg-ploiesti.ro (M.M.C.); 2 Department of Petroleum Processing Engineering and Environmental Protection, Petroleum-Gas University of Ploiesti, 100520 Ploiești, Romania

**Keywords:** additive manufacturing, polylactic acid (PLA), tribological behavior, wear mechanisms, aqueous environment

## Abstract

**Highlights:**

**What are the main findings?**

- wear behavior of 3D-printed polylactic acid (PLA) components
- surface roughness of worn surfaces;
- conductivity of aqueous friction environment.

**What are the implications of the main findings?**

- Application of low-speed polymer plain bearings;
- Identifying wear-induced degradation mechanisms

**Abstract:**

The wear behavior of 3D-printed polylactic acid (PLA) components produced by fused filament fabrication and used as friction elements in aqueous environments was investigated. Despite the growing use of additively manufactured polymers in wet systems, their wear mechanisms under such conditions remain insufficiently understood. Tests were performed under a 29 N load and 30 rpm to simulate low-speed, moderately loaded applications. PolyTerra™ PLA parallelepiped and ring specimens were analyzed through gravimetric wear testing using a Baroid lubricity tester for 135 min. During the first 105 min, both geometries showed similar mass losses, with differences below 10%. In the final stage, the parallelepiped specimen exhibited accelerated wear, while the ring specimen gained mass due to material transfer. The electrical conductivity of the medium increased significantly, from 4.6 to 1846 µS/cm, and pH rose from 7.01 to 8.04. The recovered residue matched total mass loss, and FTIR analysis confirmed the presence of PLA structures, indicating mechanical wear as the dominant process. This study provides experimental insight into the tribological behavior of 3D-printed PLA in water-lubricated conditions. By combining mass loss evaluation and medium property analysis, it improves understanding of wear mechanisms and supports the reliable design of PLA components for aqueous applications.

## 1. Introduction

Additive manufacturing technologies, particularly fused filament fabrication (FFF), have gained increasing attention due to their ability to produce components with complex geometries and tailored internal structures [1,2]. Among commonly used materials, polylactic acid (PLA) is widely adopted due to its ease of processing and low cost. However, despite these advantages, FFF-printed polymers generally exhibit limited tribological performance, including relatively high friction coefficients and reduced wear resistance, which restrict their applicability in components subjected to sliding or fluid-induced mechanical interactions.

Tribological behavior of FFF materials is influenced by both material composition and processing parameters. Numerous studies have shown that printing parameters such as layer thickness, raster angle, infill density, and orientation significantly affect wear performance [3,4,5,6,7,8]. In parallel, considerable efforts have been directed toward improving tribological properties through material modification, including the addition of graphite, natural fibers, and ceramic fillers, leading to reduced wear rates and friction coefficients [9,10,11,12,13,14,15]. While these approaches demonstrate improved performance, they often rely on complex composite systems and do not reflect the behavior of standard PLA materials commonly used in practical applications.

Furthermore, previous investigations have predominantly focused on dry sliding conditions or controlled laboratory environments. Studies on polymer components such as bearings and gears highlight the influence of operating conditions (load, speed, temperature) on wear behavior [8,16,17,18], while others analyze microstructural effects or material combinations under specific testing configurations [19]. However, limited attention has been given to the tribological response of FFF-printed PLA in aqueous or fluid-assisted environments, which are highly relevant for applications such as water transport systems.

Although polylactic acid (PLA) is widely adopted due to its biodegradability, low cost, and ease of processing, its tribological performance and long-term durability in aqueous environments remain poorly understood. Most research focuses on dry sliding conditions, composite reinforcement [20], or printing parameter optimization [21], leaving a knowledge gap about how water exposure affects friction, wear, and material degradation mechanisms. In this study, the evaluation was carried out under aqueous conditions. Unlike most existing studies conducted in dry sliding environments, this work examines PLA components operating in water-lubricated or humid conditions, which are relevant to water transport and installation systems. The scope of this study is to evaluate the wear behavior and degradation mechanisms of 3D-printed PLA components manufactured by fused filament fabrication (FFF), operating as elements of a friction coupling in an aqueous environment under conditions simulating low-speed polymer plain bearings and positioning mechanisms subjected to moderate loads and humid environments, while identifying the nature of degradation products through FTIR analysis to distinguish between mechanical wear and chemical degradation mechanisms. In contrast with other studies, the present work analyzes the wear behavior of FFF-manufactured PLA components operating in an aqueous environment and combines gravimetric wear analysis with monitoring of the physicochemical properties of the medium (electrical conductivity and pH) and FTIR characterization of the wear products. Moreover, monitoring the properties of the surrounding medium offers an indirect method for assessing the state of the tribological coupling in situations where direct evaluation of the contact surfaces is difficult or not possible, as variations in conductivity, pH, and the presence of degradation products can reflect the progression of wear and material deterioration.

Several scientific questions remain unresolved. These include the influence of water on the interaction between frictional wear and chemical degradation, such as hydrolysis, in polylactic acid (PLA). Determining how additive manufacturing-induced microstructures, including layering, porosity, and anisotropy, affect wear behavior in fluid environments is also critical. Furthermore, identifying the primary degradation pathways under combined mechanical and aqueous conditions represents a significant area of inquiry. Ad-dressing these questions is essential for advancing understanding at the intersection of tribology, polymer science, and additive manufacturing.

This research introduces several key contributions, by integrating tribological tests with chemical characterization, to elucidate the relationship between mechanical wear processes and chemical degradation. This methodology provides a more comprehensive understanding of polymer behavior in realistic service environments. Unlike most existing studies conducted in dry sliding environments, this work examines PLA components operating in water-lubricated or humid conditions, which are relevant to water transport and installation systems. We are proposing an application-oriented testing methodology by simulating real operating conditions specific to low-speed polymer plain bearings and positioning mechanisms and incorporating moderate loads and fluid interaction. Moreover, the study focuses on 3D-printed PLA without re-enforcement. While many studies improve performance through fillers or fibers, this work emphasizes the intrinsic behavior of additively manufactured PLA, highlighting its limitations and performance in real environments.

## 2. Materials and Methods

### 2.1. Preparation of the Samples

**Figure 1:**
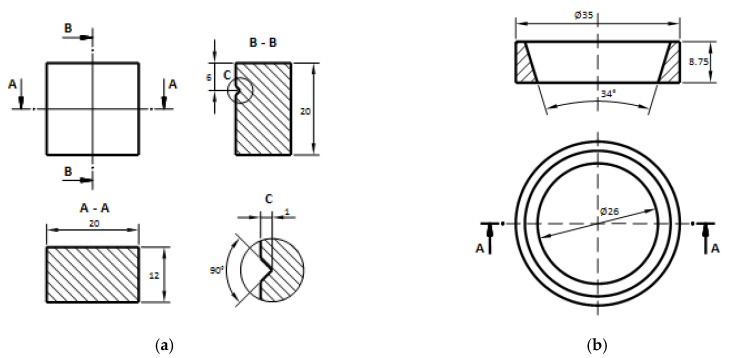
Specimens: (**a**) parallelepiped block type specimen (specimen A); (**b**) ring-type specimen (specimen B).

**Figure 2:**
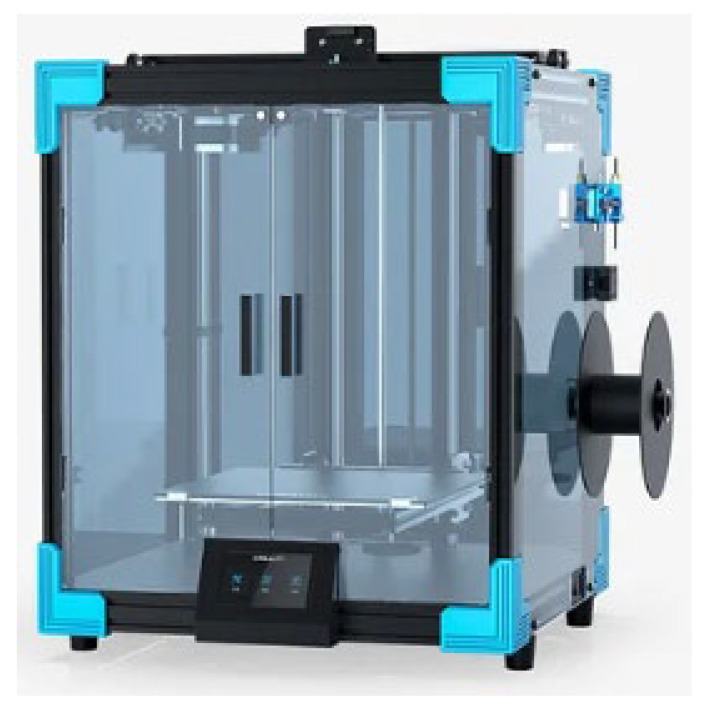
Ender-6 3D printer.

**Figure 3:**
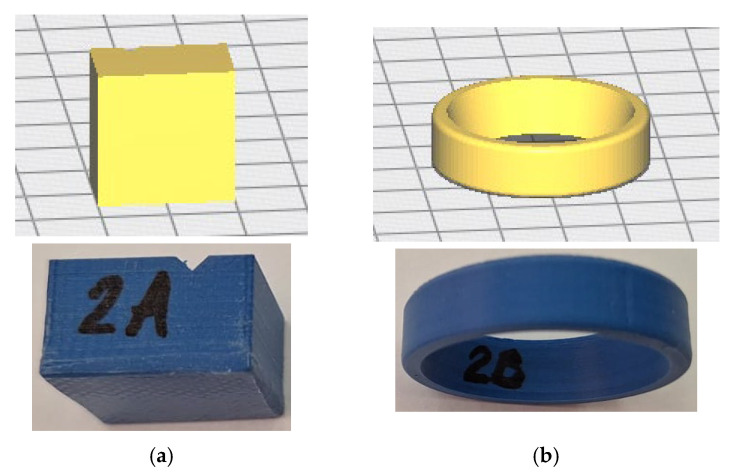
3D models of printed specimens in the slicing software and real, (**a**) specimen A; (**b**) specimen B.

The experimental investigation was conducted on a tribological pair consisting of a parallelepiped block specimen (Specimen A) and a ring-type specimen (Specimen B)—Figure 1—both manufactured from PolyTerra™ PLA (Table 1) using fused filament fabrication (FFF) technology. The specimens were fabricated with a Creality Ender-6 3D (Figure 2) printer (Creality, Shenzhen, China) using a 1.75 mm diameter PLA filament (Sapphire Blue, eSun, Shenzhen, China). Prior to fabrication, 3D models of the specimens were designed and processed using Creality Slicer 4.8.2 software (Figure 3). The printing parameters were as follows: printing speed 40 mm/s, nozzle temperature 220 °C, build plate temperature 60 °C, layer height 0.12 mm, wall thickness 1.2 mm, infill density 100%, line infill pattern, and cooling fan activated.

### 2.2. Experimental Wear Tests

Wear tests were performed using a Baroid lubricity tester (Model 3M128A, EM, Romania) under wet conditions (distilled water as working medium). The test configuration ensured a cylinder–plane linear contact between the rotating ring specimen and the stationary block specimen. The main operating parameters were: rotational speed 30 rpm, normal load 29 N, traction force 3 N (specific to low-speed polymer plain bearings or positioning mechanisms operating under moderate loads in humid environments) and a total testing time of 135 min, with measurements taken at 15 min intervals. Gravimetric wear was determined by measuring the mass loss of both specimens using an ALJ 500-4A analytical balance, accuracy 0.1 mg, (KERN & Sohn GmbH, Balingen, Germany). Due to water absorption by PLA, specimens were conditioned in a desiccator for 48 h prior to weighing to ensure complete moisture removal.

**Figure 4:**
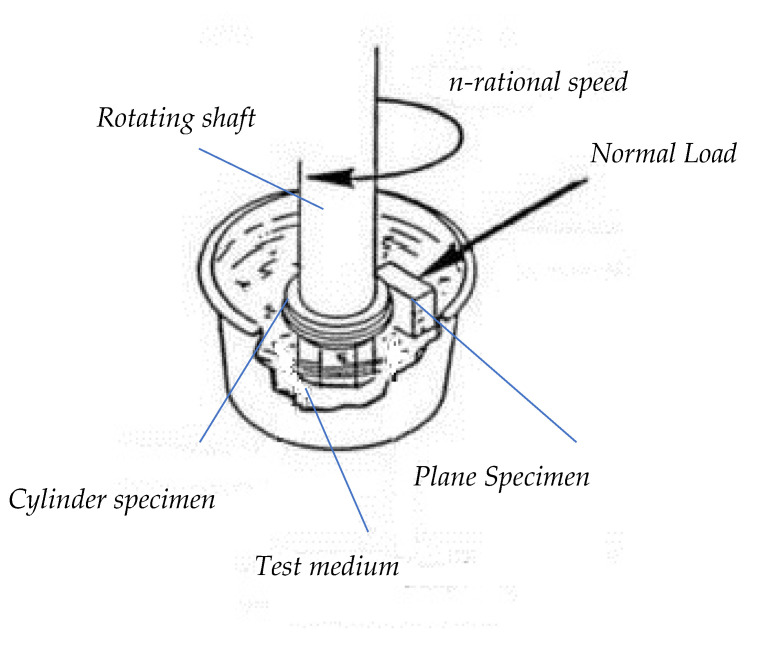
Working principal of the Baroid lubricity tester.

Figure 4 presents the loading scheme of the Baroid lubricity tester. The testing parameters were as follows:

- Loading force arm: b = 32 mm;
- Traction force arm: L = 310 mm;
- Rotational speed of the shaft (ring specimen): n = 30 rpm;
- Traction force: T = 3 N;
- Normal load: F = 29 N;
- Working medium: water.

**Figure 5:**
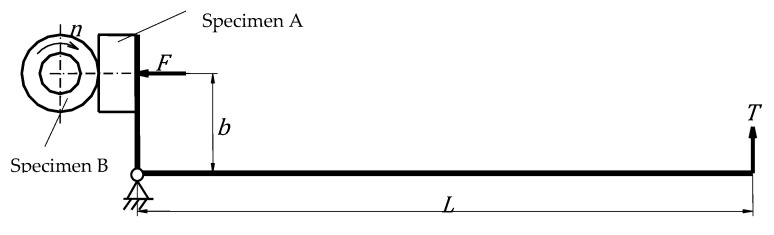
Loading scheme of the Baroid lubricity tester.

The traction force was applied using a 300 g weight (Figure 5).

Since the wear tests were carried out on a friction pair consisting of a cylindrical specimen and a parallelepiped specimen, forming a cylinder–plane linear contact, the following conditions were ensured:

- Static contact between the elements of the friction pair (no impact loading);
- Only a normal force acting along the common normal direction of the contact bodies;
- The resulting contact area (contact imprint) had a small width compared to the cylinder dimensions.

**Figure 6:**
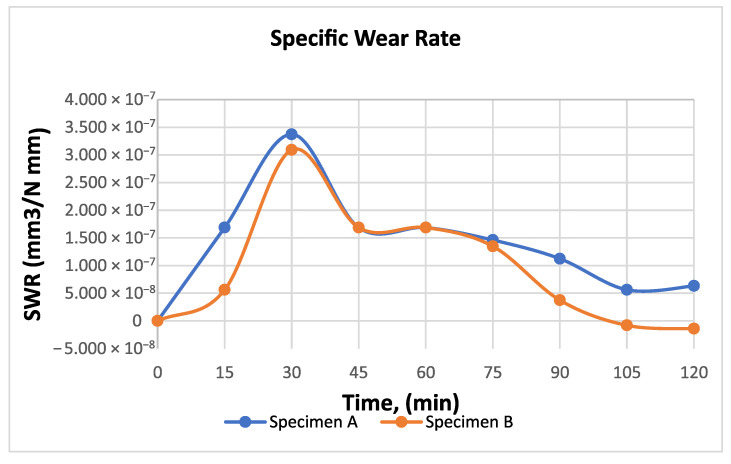
Specific wear rate of the friction pair components.

The testing methodology consisted of the following sequential steps:

- The specimens used for the experiments were initially weighed using an ALJ 500-4A precision analytical balance (Figure 5).
- The ring specimen was mounted on the rotating shaft, while the parallelepiped specimen was fixed in the movable arm holder (Figure 6).
- The Baroid apparatus was started, and with the specimens not in contact, the friction force indicator was adjusted to zero.
- With the ring specimen rotating, the two specimens were brought into contact and loaded with the predetermined normal force (the loading force was applied using calibrated weights and verified with the torque wrench of the Baroid apparatus).
- After the specified testing interval elapsed, the device was stopped and the specimens were cleaned by washing with water.
- The specimens were then placed in a sealed desiccator and maintained for 48 h to ensure complete removal of absorbed moisture.
- Each specimen was individually reweighed using the ALJ 500-4A analytical balance (weighing accuracy: 0.1 mg).

Optical microscopy observations were performed using an Olympus BX53 microscope (Olympus Corporation, Tokyo, Japan) at magnifications between 10× and 100×, 500× to analyze the worn surfaces of the specimens.

The testing cycle was subsequently resumed. Preliminary tests regarding the material behavior in the testing medium (water) showed that the material absorbs a certain amount of water, which influences the test results. For this reason, prior to weighing, the specimens were kept for 48 h in a sealed desiccator to allow the gradual removal of the absorbed moisture. This integrated methodology enabled the correlation between mechanical wear behavior and physicochemical modifications occurring in the aqueous test environment.

### 2.3. Physicochemical Characterization of the Test Medium

The test medium was analyzed before and after experiments to evaluate physicochemical changes. pH and electrical conductivity were measured using an inoLab Multi 9630 IDS multimeter (Xylem Analytics Germany Sales GmbH & Co. KG, Weilheim, Germany). Additionally, Fourier transform infrared (FTIR) spectroscopy (Shimadzu IRAffinity-1S, 380–4000 cm^−1^, 4 cm^−1^ resolution, Shimadzu Corporation, Kyoto, Japan) was performed to identify chemical species released into the medium during wear. Suspended particles generated during testing were recovered by centrifugation (Hettich ROTOFIX 32 A, 1500 rpm, 25 °C, Hettich ROTOFIX 32 A, Andreas Hettich GmbH & Co. KG, Tuttlingen, Germany), dried at 105 °C for 6 h, and gravimetrically quantified.

Since a homogeneous and uniform working medium (distilled water) was used during the experimental investigation of the wear behavior of the friction pair, samples of the medium were collected both before and after the experimental determinations in order to evaluate the interaction between the tribological coupling and the working environment.

Accordingly, the collected samples were characterized by measuring pH and electrical conductivity, using the following analytical methods:

- Determination of the conductivity and pH of the samples was performed with the inoLab Multi 9630 IDS (Germany) multimeter. The electrode used for pH was SeTix 980 with glass rod and temperature sensor. The conductivity cell was WTW IDS TetraCon 925.
- For the qualitative analysis of the materials, Fourier transform infrared spectroscopy was used to identify the functional groups present in the structures. The analysis was conducted using a Shimadzu IRAffinity-1S spectrophotometer (Kyoto, Japan), which was equipped with the GladiATR-10 accessory. The measurements were taken within the wavelength range of 380 to 4000 cm^−1^, with a spectral resolution of 4 cm^−1^.

## 3. Results

### 3.1. Wear Mechanism

Following the experimental tests, the specific wear rates values of the components of the tested friction pairs were determined, as well as the surface roughness values of the wear imprint on the parallelepiped specimen and of the ring specimen surface after each wear cycle.

**Figure 7:**
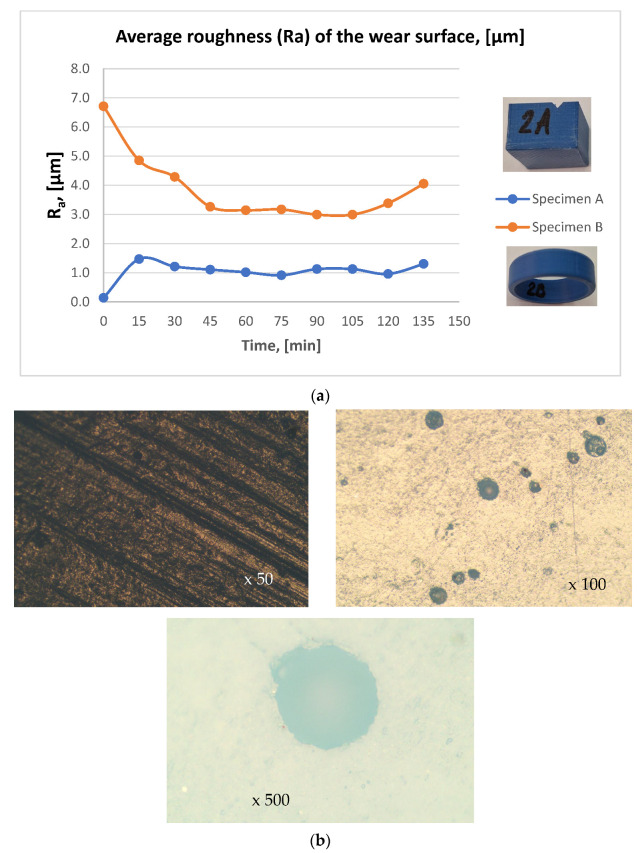
Wear mechanism: (**a**) average surface roughness (Ra) curves of the contact surfaces of the friction pair components; (**b**) microscopic examination of the contact surface imprints during testing.

Figure 6 presents specific wear rate curves of the analyzed friction pair components as a function of time, while Figure 7 shows the diagrams of the average roughness of the contact surfaces of the examined friction pair components.

The total time considered for the interpretation of the measurement results was 135 min.

Based on the experimentally determined values for the two specimens of the analyzed friction pair, the following observations were made:

- The evolution of the specific wear rate (SWR) for both specimens shows an initial increase, reaching a maximum at approximately 30 min, corresponding to the running-in stage. After this period, the wear rate decreases and stabilizes between 45 and 75 min, indicating more stable contact conditions. In the final stage of the test, specimen B exhibits a significant reduction in SWR, approaching near-zero values, which can be associated with material transfer or adhesion from specimen A. Overall, specimen A maintains slightly higher wear rates during most of the experiment (Figure 6). During the 105–135 min interval, the parallelepiped specimen exhibited an accelerated increase in SWR, while the ring specimen showed a corresponding gain in mass, as reflected by the descending trend of its wear curve; this behavior clearly indicates the occurrence of material transfer within the friction coupling, with debris from one component adhering to the counter-face surface.
- In the 0–30 min time interval, a sharp increase in SWR is observed for both specimens, corresponding to the running-in period of the material.
- The running-in stage is also reflected in the evolution of the contact surface roughness curves of the two components: the parallelepiped specimen exhibits a pronounced increase in contact surface roughness, whereas the ring specimen shows a significant decrease in contact surface roughness.
- Analyzing the contact surface roughness of the two components in the 105–135 min interval, a marked increase in roughness is observed for the ring specimen compared to the parallelepiped specimen, whose roughness, except for slight variations, tends to stabilize (Figure 7).
- The variations in contact surface roughness, correlated with the weight loss/gain trends during the final testing period, indicate a tendency toward seizure of the tribological pair.
- Microscopic examination of the contact surface imprints after testing revealed the presence of pore structures. These pores may positively influence the coupling’s performance, as their filling with the working medium (e.g., water, oil) can facilitate lubrication between the contact interfaces.

### 3.2. Characterization of the Test Medium

The experimental values obtained from the tests performed on the working medium are presented in Table 2.

Initially, the medium used for the friction tests, namely water, exhibited very low electrical conductivity (4.6 µS/cm), as water molecules lack free charge carriers capable of sustaining electrical conduction. After the tribological tests, a pronounced increase in electrical conductivity was observed, reaching 1846 µS/cm. A precise definition of the test medium is essential. The experiments employed distilled water, which initially exhibited very low ionic content, resulting in a low baseline conductivity. Under these conditions, even small quantities of dissolved species can produce significant increases in conductivity.

The observed increase in conductivity is attributed to several concurrent mechanisms. Wear-induced material release during friction causes mechanical degradation of polylactic acid (PLA), generating micro- and nanoscale debris. Although bulk PLA is electrically insulating, its degradation yields low-molecular-weight compounds, such as lactic acid and oligomers, which partially dissolve in water and contribute ionic species.

When water is used as the test medium, hydrolytic degradation occurs under tribological conditions. The combined effects of mechanical stress, frictional heating, and water exposure accelerate PLA hydrolysis, resulting in the formation of carboxyl- and hydroxyl-containing compounds that dissociate and increase ionic conductivity.

Two additional factors warrant emphasis: the release of additives and impurities, as well as the potential contribution from counter-face materials. Commercial PLA filaments may contain residual monomers, catalysts, plasticizers, or pigments that can leach into the aqueous medium during testing, thereby significantly increasing conductivity.

Given the medium’s initially ultra-low conductivity of approximately 4.6 µS/cm, the increase to approximately 1846 µS/cm, while substantial, is physically plausible, particularly in the presence of accumulated degradation products and impurities.

The increase in conductivity does not arise from the intrinsic electrical properties of PLA, but rather from tribological and chemically induced transformations that release ionic and polar species into the aqueous medium. This increase is attributed to the partial solubilization of compounds originating from the chemical structure of the friction coupling components during the wear process. In the presence of such dissolved species, the test medium becomes a significantly better electrical conductor.

Although polylactic acid (PLA) is inherently electrically insulating, its degradation products or suspended fragments can influence the electrical conductivity of water, depending on their concentration and chemical composition. Moreover, PLA, in filament form, may contain residual monomers, additives, or processing-related impurities, which can be released into the aqueous medium during tribological loading and subsequently contribute to the observed increase in conductivity. These findings support the hypothesis that the conductivity rise is associated with wear-induced material release rather than intrinsic electrical properties of the polymer.

**Figure 8:**
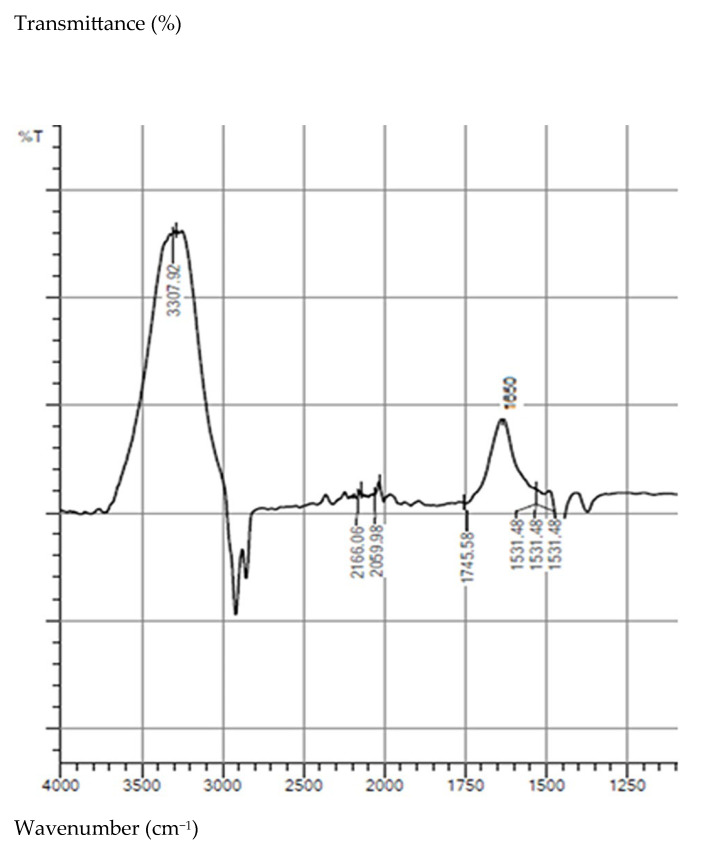
FTIR spectrum of water after the wear test.

The analysis of the test medium recovered at the end of the experiments revealed the presence of solid particles in suspension, originating from the degradation of the friction coupling components. The suspended particles were recovered by centrifugation at 25 °C and 1500 rpm, using a Hettich ROTOFIX 32 A centrifuge. The collected suspension was subsequently dried in an oven at 105 °C for 6 h until constant mass was achieved. The total mass of the recovered solid was 0.0169 g, relative to 200 mL of test solution. This value falls within the range of cumulative mass losses measured for the two samples, as indicated by the wear curves of the friction coupling components (Figure 8).

The presence of these particles correlates with the increase in the electrical conductivity of the medium observed after the tribological tests. Although the experimental data do not allow the exact mechanism of this correlation to be determined, it is reasonable to assume that the fine wear particles contribute indirectly to the increase in conductivity, either through the limited release of ionic species or by increasing the effective surface area available for interaction with the liquid medium.

The small particle size is suggested by the stability of the suspension and the requirement of centrifugation for particle separation, indicating a fine, likely micro- or submicrometric, size range. FTIR analysis of the recovered test medium highlights absorption bands characteristic of the materials composing friction coupling, without the appearance of new, well-defined signals associated with major chemical degradation products. These results indicate that the deterioration process is dominated by mechanical wear mechanisms, while chemical degradation plays a secondary role.

FTIR spectrophotometric analysis in the mid-infrared region of 4000–500 cm^−1^ of the water sample after testing confirmed the presence of structures analogous to those found in the material composing the two friction coupler samples (Figure 8).

The results of the FTIR analysis, illustrated in Figure 8, reveal that the stretching vibrations observed at 3307 cm^−1^ are associated with the O-H bonds from the PLA structure. During the tests, degradation processes of PLA may also occur. Their presence is indicated by the appearance of a low-intensity peak at 1531 cm^−1^, attributed to carboxylate (COO^−^) groups, particularly those formed during the degradation of polylactic acid (Table 3).

Confirmation of the presence of PLA residues in the recovered water sample is provided by the appearance of the band at ~1745 cm^−1^, which is characteristic of the carbonyl (C=O) stretching vibrations of PLA. Also, the FTIR spectra indicates the presence of structures originating from the dyes present in the material from which the two friction coupler samples were made (2166 and 1650 cm^−1^).

## 4. Discussion

In study [23], wear and worn surfaces were analyzed referring to ABS material parts and graphene-enriched ABS in wet conditions. Surface morphology was investigated, and wear track analysis was conducted. The results revealed an increase in surface roughness within the worn regions.

A comparative study was conducted on ABS and PA 6, with CF reinforcement of 3D-printed parts [24], where samples were also socked in distilled water, water with 20% NaCl, and dry conditions, and it was proved that the exposure to the distilled water environment rendered the ABS-based components susceptible to accelerated wear.

The evolution of the specific wear rate (SWR) of the two specimens as a function of time is presented in Figure 6. At the beginning of the test, both specimens exhibit a gradual increase in wear rate, reaching a maximum at approximately 30 min. The peak SWR values are about 3.3 × 10^−7^ mm^3^/N·mm for specimen A and 3.0 × 10^−7^ mm^3^/N·mm for specimen B, indicating an initial running-in stage characterized by higher material removal, values that can be compared with neat PLA 3.04 × 10^−8^ mm^3^/N·mm at 10 N load and 1 m/s sliding speed, whereas a maximum specific wear rate of 32 × 10^−8^ mm^3^/N·mm was observed at 30 N load and 3 m/s sliding speed [25].

After this stage, the wear rate of both specimens decreases and stabilizes between 45 and 75 min, suggesting the establishment of more stable contact conditions. In the later stage of the test, the SWR continues to decrease, particularly for specimen B, which even approaches near-zero or slightly negative values around 105–120 min. This behavior may indicate material transfer or adhesion from specimen A to specimen B, which is consistent with the mass variation observed during the experiments.

Overall, specimen A maintains slightly higher wear rates during most of the test, while specimen B exhibits lower wear in the final stage, likely due to the accumulation of transferred material on its surface and the different thermal and contact conditions associated with the rotating geometry. Although the geometries do not directly exploit the design freedom of additive manufacturing, their selection was dictated by the requirements of the standardized block-on-disc tribological testing Baroid configuration, which imposes specific constraints on specimen shape and dimensions. At the same time, the obtained results reflect the intrinsic material and process-related behavior of FFF-printed PLA. Therefore, the results drawn from this study can be extended to components with more complex geometries, as the wear mechanisms are primarily governed by material properties and printing parameters rather than the overall shape. The selection of fused filament fabrication (FFF) for manufacturing PLA components in water-related tribological applications is strongly justified by both functional benefits and research significance.

First, FFF enables the fabrication of geometrically complex, application-specific components, including polymer bushings, plain bearings, and elements integrated into fluid transport systems. In water installations, components such as valve supports, guide elements, or low-load-bearing interfaces often require custom geometries, internal channels, or rapid replacement. These requirements are challenging and costly to address using conventional manufacturing techniques.

Second, FFF inherently produces parts with layered microstructures, anisotropy, and controlled porosity, all of which significantly influence tribological behavior. These characteristics are not defects but essential features that require a thorough understanding, particularly in aqueous environments where water may penetrate interlayer regions or degrade interfacial bonding over time. Additionally, friction and wear mechanisms can differ substantially from those observed in bulk materials.

Therefore, investigating FFF-printed PLA is both practical and scientifically necessary, since its behavior cannot be directly extrapolated from that of injection-molded polymers.

Another justification for selecting FFF is its status as one of the most widely adopted additive manufacturing techniques for thermoplastics such as PLA, which enhances its relevance for real-world engineering applications. Its accessibility, low cost, and material efficiency make it particularly suitable for decentralized production of spare parts, rapid prototyping and iteration, and on-demand manufacturing in infrastructure systems, including water networks.

From a sustainability perspective, combining FFF with PLA addresses the growing demand for environmentally responsible engineering solutions. However, this approach also presents challenges related to hydrolytic degradation and durability in humid or submerged conditions, which require systematic evaluation.

The use of FFF in this study is intended to cover a clear research gap. While many studies focus on optimizing printing parameters or enhancing materials with fillers, limited knowledge exists regarding the performance of as-printed PLA components under simultaneous mechanical loading and water exposure. Investigating FFF-produced parts under these conditions yields insights that are directly applicable to practical engineering scenarios rather than idealized material behavior.

FFF serves not only as a convenient manufacturing method but also as a critical variable influencing material performance. Its selection is essential for accurately assessing the tribological reliability of PLA components in water-based systems.

From an application perspective, FFF provides significant design flexibility and rapid manufacturability, facilitating the production of customized components for water systems without the need for molds, specialized tooling, or complex subtractive processes. In contrast, conventional methods such as injection molding incur high initial tooling costs, are inefficient for small batch production or prototyping, and do not readily support design iteration or geometry optimization.

Consequently, FFF is particularly well-suited for producing low-volume, application-specific parts, such as polymer elements used in water installations, including guides, bushings, or positioning components, where customization and rapid replacement are critical.

Furthermore, the use of FFF is scientifically justified because it produces components with unique micro-structural characteristics, such as interlayer interfaces, anisotropy, and voids, which are not present in conventionally manufactured parts. These features significantly affect friction and wear behavior, facilitate preferential pathways for water ingress, and can accelerate mechanical degradation and hydrolysis in materials such as polylactic acid (PLA).

Investigation of FFF-produced components is essential because their performance cannot be reliably inferred from data on bulk or injection-molded materials. The selection of simple geometries, such as flat or basic contact surfaces, is both intentional and supported by scientific rationale. Employing simple geometries ensures controlled, reproducible tribological conditions, facilitating the isolation of key variables, including load, sliding speed, and environmental factors. In contrast, complex geometries introduce non-uniform contact stresses, variable lubrication regimes, and additional sources of experimental uncertainty. Furthermore, the use of simple geometries enables clear interpretation of wear mechanisms, allowing for distinction between abrasive and adhesive wear, as well as mechanical and chemical degradation, as validated by FTIR analysis.

This approach is consistent with fundamental research in tribology, where simplified contact configurations are commonly used to establish baseline material behavior before investigating more complex systems. By minimizing geometric complexity, the study can directly assess the influence of the fused filament fabrication (FFF) process and the intrinsic properties of polylactic acid (PLA) in aqueous environments, which constitute the primary focus of the research.

Polylactic acid (PLA) is widely recognized as an electrical insulator. However, during mechanical testing in aqueous environments, compounds within friction couplings may undergo decomposition, leading to an increase in the electrical conductivity of the test medium [26,27,28]. These degradation processes can occur even at ambient temperatures and are promoted by delamination, shear stresses, and localized temperature rises generated during friction testing [28,29,30,31,32]. Consequently, scission of aliphatic ester chains, such as those derived from lactic acid, may take place, accompanied by hydrolysis and depolymerization of the polymer matrix [32,33,34].

In the mechanisms of PLA degradation in the presence of water, the first step is the hydrolytic degradation of the compounds originating from the chemical structure of the friction coupling components. The acidic products formed during the tribological process can accelerate the degradation step. During this stage, low-molecular-weight intermediates are produced. Between them are oligomers and lactic acid monomers. In the second step, a partial solubilization can occur. Other compounds identified are acetic acid, pyruvic acid, or some short oligomeric lactic acid chains with terminal –COOH groups. These carboxylic groups can have an electrical conductivity ranging from 102–104 µS/cm, depending on the concentration of acidic degradation products in water. The acidic degradation products increase conductivity through ionic conduction at the surface of the specimens.

Moreover, in the composition of the polymeric specimens, additives, dyes, or filler with high electric conductivity like carbon black (105–108 µS/cm) or graphene (until 109 µS/cm) [35,36] are used. In that context, the tribological media become a weak electrolyte system, with electrical conductivity between 102 and 105 µS/cm.

In the presence of such dissolved species, the test medium becomes a significantly better electrical conductor.

At this stage, soluble degradation products, such as oligomers, carboxylic end groups, and ionic species resulting from the partial dissociation of lactic acid, can be detected in the surrounding medium [37], as illustrated by the following equilibrium reaction:

CH_3_CH(OH)COOH + H_2_O ⇌ H_3_O^+^ + CH_3_CH(OH)COO^−^ (1)

Reaction (1) represents the acid dissociation of lactic acid in water. This equilibrium process occurs readily in aqueous solutions without the need for a catalyst. An increase in temperature, for example, due to friction during tribological contact, can enhance the degree of dissociation and promote the formation of ionic species. The release of hydrogen ions (H^+^) and lactate anions increases the solution’s ionic strength, thereby contributing to the observed rise in electrical conductivity.

Furthermore, previous studies have shown that prolonged friction testing of PLA-based components enables monitoring of electrical conductivity in the test medium as a function of time, thereby providing an indirect method for assessing the extent of coupling degradation [38]. An increase in conductivity during testing can therefore be correlated with progressive material breakdown. In addition, colorants, pigments, and stabilizing additives incorporated during the manufacturing of friction couplings may leach into the aqueous environment and form ionic species, further contributing to the measured conductivity.

Fourier transform infrared (FTIR) spectroscopy is commonly employed to identify degradation products in aqueous media [34,39,40,41]. Hydrolytic degradation is indicated by changes in the intensity of the O–H stretching bands observed in the range of 3402–3667 cm^−1^, which are associated with hydroxyl groups formed during PLA chain scission. The literature also reports structural modifications arising from the cleavage of saturated aliphatic chains and from oxidative processes, evidenced by the formation of additional carboxyl groups and corresponding alterations in characteristic carbonyl absorption bands [33,41].

FTIR spectrophotometric analysis was performed on the aqueous solution containing solubilized degradation products of polylactic acid. Comparison between the obtained spectrum and reference spectra reported in the literature [40,42] shows the presence of a band at approximately 1745 cm^−1^, characteristic of the carbonyl (C=O) stretching vibration of PLA. In the analyzed aqueous solution, this band appears significantly less intense, indicating partial degradation of the polymer and the formation of lower-molecular-weight species.

Additionally, several absorption bands are observed in the 1530–1250 cm^−1^ region, which can be attributed to asymmetric and symmetric C–H bending vibrations associated with PLA fragments and related degradation products [40,42].

These spectral features confirm the presence of PLA degradation compounds in the aqueous medium.

Future research will focus on wear testing in other environments, like oil or saline solutions, to highlight the PLA-printed parts wear behavior in different conditions and the suitability of the parts in machine parts specific to a wide range of industries.

## 5. Conclusions

1. The conducted experiments contribute to the evaluation of the durability of friction coupling components manufactured from PLA thermoplastic materials.
2. The wear curves of the two tested specimens show similar wear behavior. Toward the end of the test period, specimen B exhibited a slight mass increase and higher surface roughness, which is attributed to material transfer and adhesion from specimen A.
3. The deterioration of the tribological pair was also reflected by changes in the physicochemical properties of the testing medium. Electrical conductivity increased significantly from 4.6 µS/cm to 1846 µS/cm, indicating the presence of wear debris and additional ionic species generated during the test.
4. In contrast, the pH showed only minor variation, increasing slightly from 7.01 to 8.04, suggesting that chemical degradation was limited and that the processes occurring were mainly mechanical.
5. FTIR analysis confirmed the presence of PLA-related structures in the medium, indicating the solubilization of some degradation products. These observed bands reflect the release of material fragments during the wear process. Overall, the results show that the degradation of the tribological coupling is dominated by mechanical wear with limited chemical interaction with the aqueous environment.
6. The study bridges the gap between laboratory-scale tribological testing and real-world application conditions, providing new insights into the durability and reliability of FFF-manufactured PLA components used in water-related systems.

## Figures and Tables

**Table 1:** Characteristics of the filament.

Type	PolyTerra^TM^ PLA
Filament Diameter	1.75 mm
Color	Sapphire Blue
Printing Speed	30–70 mm/s
Printing Temperature	190–230 °C
Bed Temperature	25–60 °C

**Table 2:** Properties of the working medium (distilled water).

Property	Unit	Initial Value	Final Value	Analysis Method
pH, at 25 °C	–	7.01	8.04	SR EN ISO 27888:1997 [22]
Conductivity, at 25 °C	µS/cm	4.6	1846	SR EN ISO 27888:1997 [22]

**Table 3:** Functional groups in vegetable oils related to FTIR spectra.

Wavenumbers (cm^−1^)	Functional Group	Mode of Vibration	Possible Provenance	Absorption Intensity
3307	O-H	Stretching	PLA	strong
2166	C≡N	Stretching	Dyes	weak
1745	C=O	Stretching	PLA	weak
1650	N-H	Bending	Dyes	strong
1531	(COO^−^)	Antisymmetric stretching	Degradation of PLA	weak

## Data Availability

The original contributions presented in this study are included in the article. Further inquiries can be directed to the corresponding authors.

## Abbreviations

The following abbreviations are used in this manuscript:

FDM	Fused deposition modeling
PLA	Polylactic acid
ABS	Acrylonitrile butadiene styrene
PA	Polyamide
FTIR	Fourier transform infrared spectroscopy
